# Augmented Reality-Guided External Ventricular Drain Placement: A Case Report

**DOI:** 10.7759/cureus.64403

**Published:** 2024-07-12

**Authors:** Andrew Janssen, Arthur Wang, Aaron S Dumont, Johnny Delashaw

**Affiliations:** 1 Department of Neurological Surgery, Tulane University School of Medicine, New Orleans, USA; 2 Department of Neurosurgery, Tulane University School of Medicine, New Orleans, USA

**Keywords:** neurotechnology, hydrocephalus, neurosurgery, mixed reality (mr), augmented reality, external ventricular drain (evd), ventriculostomy, neurocritical care unit

## Abstract

The placement of an external ventricular drain (EVD) is a critical neurosurgical procedure used to relieve intracranial pressure in patients with conditions such as hydrocephalus, traumatic brain injury, and intracranial hemorrhage. Traditional methods rely heavily on anatomical landmarks and the surgeon's experience, which can lead to variability in outcomes and increased risk of complications. Neuronavigation, while available, is infrequently used due to the size, cost, and set-up times associated with these devices. This report explores the use of a headset-based augmented reality (AR) system for guidance during the EVD placement procedure. We describe an AR system that overlays a 3D model of the patient’s cranial anatomy, derived from preoperative imaging, onto the patient's head. This system is a head-mounted display and utilizes a rapid fiducial-less registration to provide the surgeon with visualization of 3D anatomy, and targeted trajectories. The system was used with a 32-year-old patient undergoing EVD placement prior to a cranioplasty. Due to the atypical cranial anatomy and due to prior procedures and midline shift, this relatively high-risk catheter placement was an ideal circumstance for the use of AR guidance during the EVD placement. This report described an early use of AR for EVD placement and represents a substantial advancement in neurosurgical practice, offering enhanced precision, efficiency, and safety. Further large-scale studies are warranted to validate these findings and explore the broader applicability of AR in other neurosurgical procedures.

## Introduction

The integration of advanced technology in medical procedures has significantly enhanced the precision, safety, and outcomes of various treatments. One such technological innovation is augmented reality (AR), which is increasingly being explored and utilized in neurosurgery [1-3]. Specifically, AR due to its unique 3D views, small footprint, and improved processing speeds is an ideal solution for traditionally non-navigated procedures such as external ventricular drain (EVD).

EVD placement, a critical neurosurgical procedure, involves inserting a catheter into the brain's ventricular system to relieve intracranial pressure by draining cerebrospinal fluid [4-7]. Despite being routine, this procedure is not without risks, including misplacement of the catheter, which can lead to serious complications and the need for additional interventions. The procedure relies heavily on the surgeon's experience and anatomical knowledge and is traditionally performed without navigation due to the large size, and long setup times required of currently available solutions, and is frequently done at the bedside which further precludes current neuronavigation use [6].

AR offers a transformative approach to EVD placement by providing surgeons with enhanced views of the patient's anatomy. AR technology overlays digital information, such as 3D reconstructions of imaging data, directly onto the surgical field [8,9]. This allows for improved visualization of the ventricles and critical surrounding structures, facilitating more accurate and efficient catheter placement. The key, however, is providing this visualization in a manner that integrates seamlessly into the clinical workflow with minimal setup time and a small footprint in the procedure room. By augmenting the surgeon's natural vision with virtual elements, AR can potentially reduce the margin for error, minimize invasive maneuvers, reduce the procedural learning curve, and improve patient outcomes. We aimed to describe, to the best of our knowledge, the first documented use of a novel small-footprint AR system for 3D visualization and guidance during an EVD placement.

## Case presentation

**Figure 1:**
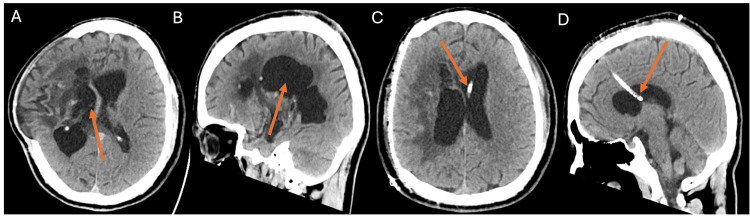
Preoperative and postoperative CT imaging (A, B) Axial and sagittal views of the preoperative patient computed tomography (CT) scan. Arrows point to the lateral ventricles, demonstrating ventriculomegaly. (C, D) Axial and sagittal views of the postoperative patient CT scan. Arrows point to the external ventricular drain catheter.

A 32-year-old man was admitted for a right cranioplasty after undergoing a right decompressive hemi-craniectomy for intracranial hemorrhage from a ruptured right basal ganglia high-grade arteriovenous malformation. Nine months following his hemi-craniectomy, he was admitted for right cranioplasty. Upon workup, the patient was found, however, to have persistent, asymmetric ventriculomegaly with continued parenchymal protrusion through the calvarial defect. The patient also had a prior contralateral burr hole and incision scar in suboptimal placement from the prior trauma procedure, further complicating the use of standard, non-navigated landmarks and trajectories. The decision was made to place an EVD prior to cranioplasty for relaxation. Due to the atypical presentation described, this relatively high-risk catheter placement was an ideal circumstance for the use of AR guidance during the EVD placement. The patient’s head was not pinned and his 3D anatomy was registered onto the head through a headset display allowing the surgeon to visualize the ventricles, midline, and suture lines. A virtual trajectory line was placed by the surgeon using the prior entry point/incision and an appropriate target. The surgeon aligned the ventricular catheter with the virtual trajectory line during insertion and successfully cannulated the ventricles in a single pass, with successful drainage and relaxation of the defect. Postoperative CT imaging confirmed proper placement of the left frontal ventricle (Figure 1).

AR technology platform

A headset-based AR system was incorporated into the EVD workflow. The software (Hoth Intelligence, Philadelphia, Pennsylvania) functions on the Microsoft Hololens 2 head-mounted display (HMD) (Redmond, Washington) [10,11]. The Microsoft Hololens 2 is an untethered optical see-through HMD that displays digital content (i.e., holograms, images, screens) onto the users’ real-world field of view. The entire system operates out of the headset and does not require external cameras, computers, or processing towers.

AR registration

**Figure 2:**
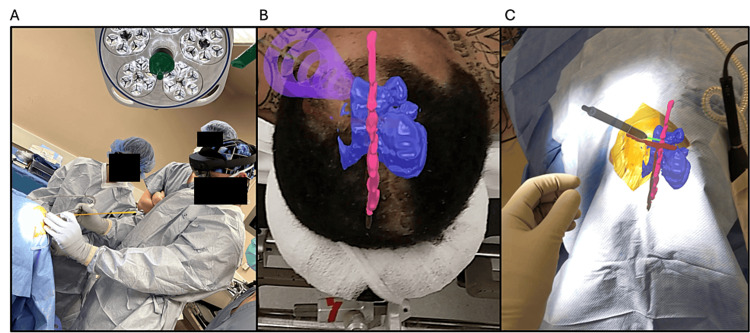
Augmented reality (AR) visualization of registered three-dimensional ventricular anatomy (A) Surgeon wearing the augmented reality (AR) system during the placement of the external ventricular drain (EVD). (B) Surgeon's view through the headset displaying a three-dimensional (3D) model of ventricles, trajectory line, and midline. (C) Surgeon's view through the headset after draping, showing a 3D model of ventricles, midline, and coronal sutures.

The AR system registers 3D models of the patient’s anatomy onto the head via a fiducial-less registration process. The system employs surface matching and feature detecting to align the 3D model with the patient based on landmarks of the head. In this case, the patients’ model consisted of four components including face, ventricles, midline, coronal, and sagittal sutures. The addition of a small infrared (IR) sphere array after registration maintains the accuracy of the registered 3D model throughout the movement of the patient’s head. The 3D model was registered onto the patient’s head prior to starting the procedure. To register, the surgeon looks at the patient’s face while wearing the headset. The registration workflow was completed in 17.2 seconds. Once the 3D model was registered with the head, the surgeon was able to map out a virtual trajectory towards the intraventrical target which was then used to position the physical catheter during insertion (Figure 2).

## Discussion

The advent of AR represents a significant advancement in the field of neurosurgery. In particular, AR is suited to advance the safety and efficacy of currently non-navigated procedures such as EVD placement. This notion is substantiated by prior studies that have demonstrated the efficacy of AR for EVD placement in cadaveric or phantom studies [12-14]. Traditional neuronavigation systems, while effective, often require substantial infrastructure, including dedicated space, extensive hardware, and complex set-up procedures. These requirements can limit their utility in urgent and resource-constrained settings. As such, procedures such as EVD placement or subdural drain placement are traditionally done without image guidance and thus are error-prone. In contrast, AR systems have a small operating footprint and are designed to be compact, portable, and user-friendly, making them particularly suitable as visualization solutions for non-navigated procedures.

We describe the use of a novel AR system for guidance during an EVD placement in a patient undergoing cranioplasty. The procedure described here was complicated by the presence of distorted ventricular anatomy and midline shift and thus we chose to include the AR system to provide visualization of 3D anatomy overlaid onto the patients’ head. While wearing the headset, the surgeon was able to visualize the ventricular anatomy as well as midline, coronal sutures, and trajectories. One of the primary advantages of the system is its accessibility and convenience. The system registers without the need for fiducial markers and thus the imaging protocol for the patient doesn’t need to be changed to accommodate the technology. Due to its small size, the systems can be quickly deployed at the patient's bedside, providing guidance without the need to transfer less mobile traditional navigation to the site of service. Lastly, the fast registration speed allows for access to 3D visuals without a disruption of the clinical workflow. In this case, despite midline shift and irregularly shaped ventricles, the ventricles were successfully canulated on the first pass. There have been various other reports describing the clinical use of AR in neurosurgery for procedures such as pedicle screw placement or preoperative planning tumor resection surgery; however, the clinical demonstration of the use of AR for EVD placement is novel. Various studies have explored this application in cadaveric models; however, this is to our knowledge, the first description of patient use of a head-mounted AR display for guidance during EVD placement [12-14].

There are challenges that must be considered in order to drive greater adoption of AR. AR systems display digital models in the surgical field. This may be distracting for some users; however, the system was built to allow for voice-controlled adjustments to image display such as hide/show or transparency toggles in order to customize the users’ display. Furthermore, the requirement to wear a headset may be unappealing to surgeons and thus studies evaluating the ergonomics and comfort of the system are warranted. Lastly, others have raised concerns about AR system's ability to transmit data safely and securely with low latency [15]. It is plausible to most that enhanced visualization of registered 3D anatomy has safety benefits during EVD placement. However, if this technology is to become the standard of care for EVD placement, more work demonstrating superiority, clinical efficacy, and ease of use is warranted.

AR technology represents an opportunity to change the standard of care in neurosurgery. The ability to visualize 3D anatomy for procedures has tremendous advantages over the traditional freehand approach to this procedure. For junior residents or advanced practice providers who may not have mastered the spatial interpretation of 2D imaging, the 3D view of AR can significantly lower the learning curve for this procedure [16-18]. Additionally, the display of key features such as suture lines, midline, and intraventricular targets represents a quality and safety advantage that is not as available when the procedure is performed blindly. Therefore, this clinical work represents an innovation in the field and will likely drive greater exploration and adoption of mixed reality technologies. While this report focuses on the application for EVD, the system has tremendous potential for other neurosurgical procedures including ventriculoperitoneal shunt, cortical-based tumors, intracerebral hemorrhage, and subdural hematoma evacuation. Future studies exploring these applications are warranted.

## Conclusions

This case describes the use of a small footprint, fiducial-less AR system for the placement of an EVD. The system enabled the surgeon to visualize ventricular anatomy and trajectory lines overlaid onto the patient's head throughout the procedure. To this day, EVD placements are predominantly placed without image guidance due to the inconvenience of integrating traditional navigation systems into the clinical workflow. The size and speed of a system represent a promising solution to the lack of advanced visualization during EVD placement. Despite the clear advantages, challenges remain in ensuring the consistency, reliability, and seamless integration of AR systems into existing medical workflows. Ongoing research and development are essential to address this and to explore new applications and innovations in AR for neurosurgery. AR is set to play a pivotal role in the future of neurosurgery, revolutionizing the way procedures are performed and enhancing the capabilities of surgeons. As technology continues to evolve, AR will undoubtedly contribute to safer, more efficient, and more effective neurosurgical care, ultimately improving the lives of patients worldwide.
